# Association between obesity, common chronic diseases and health promoting lifestyle profiles in Hong Kong adults: a cross-sectional study

**DOI:** 10.1186/s12889-020-09726-x

**Published:** 2020-10-28

**Authors:** Yan Sin Leung, Jack Jock Wai Lee, Maria Ming Po Lai, Chole Kei Ming Kwok, Ka Chun Chong

**Affiliations:** Jockey Club School of Public Health and Primary Care, The Chinese University of Hong Kong, Hong Kong, China

**Keywords:** HPLP-II, Lifestyle, Chronic disease, Obesity, Chinese adults, ARIA

## Abstract

**Background:**

Recent surveys revealed that the health status of many people from Hong Kong is far from ideal. Although non-communicable diseases are largely preventable, few relevant health promotion and disease prevention programs are available. Thus, we assessed the health indicators of Chinese adults in Hong Kong to investigate the relationship between obesity, common chronic diseases, and health-promoting lifestyle profiles to provide inspirations for decision makers in formulating targeted disease prevention and health management programs.

**Methods:**

This is a secondary analysis of a data set of 270 community-dwelling Hong Kong adults who were within the eligible age range between 18 and 80 years without eye diseases that affect retinal photographs. The study exposure variable, health-promoting lifestyle profiles, was measured using the Health-Promoting Lifestyle Profile II (HPLP-II) questionnaire. The primary outcome variable, obesity, was defined using body mass index and waist-hip ratio. The secondary study outcome, estimated chronic diseases, including of anemia, chronic kidney disease, and cardiovascular disease, were estimated using automatic retinal image analysis from the retinal images. Data were analyzed using tests of proportion, the independent sample t-tests, Welch’s t-test, and binary logistic regression models.

**Results:**

All HPLP-II subscales had positive responses (≥ 2.5). Significant differences were noted between men and women in the health responsibility and nutrition subscales (Health Responsibility: *p* = 0.059; Nutrition: *p* = 0.067). Regression models revealed that nutrition (adjusted odds ratio [AOR] = 0.41; *p* = 0.017), physical activity (AOR = 0.50; *p* = 0.015), interpersonal relations (AOR = 2.14; *p* = 0.016), and stress management (AOR = 2.07; *p* 0.038) were associated with obesity; while spiritual growth (AOR = 0.24; *p* = 0.077) and interpersonal relations (AOR = 5.06; *p* 0.069) were associated with estimated chronic kidney disease.

**Conclusions:**

Improving health behaviors may control or alleviate the prevalence of obesity and chronic kidney disease. These findings could arouse concern about lifestyle behaviors and promote self-assessment of health-promoting lifestyles to the general public. The study also provided new insights into the relationship between the HPLP-II and other common chronic diseases that warrant further study.

**Supplementary Information:**

The online version contains supplementary material available at 10.1186/s12889-020-09726-x.

## Background

Non-communicable diseases (NCDs) are not only major causes of ill-health and disability but also a top killer worldwide, accounting for 71% of deaths annually [1]. The incidence and prevalence of chronic diseases in Hong Kong are showing worrying trends. In the Population Health Survey (PHS) 2017, 50% of participants were classified as overweight or obese [2]. Moreover, in the Thematic Household Survey Report No. 63, nearly one-third of the Hong Kong population (1,983,200 persons, 28.4%) was diagnosed with chronic diseases [3]. In particular, a large proportion of this population has hypertension (13.7%), followed by high cholesterol (7.2%), diabetes (5.8%) and coronary heart disease (2.1%) [3].

NCDs are largely preventable and highly manageable by modification of the associated risk factors, many of which are included in lifestyle, such as physical inactivity [4]. Therefore, health promotion and disease prevention strategies should be developed to alleviate this situation. Although several studies have revealed an association between lifestyle and certain diseases such as type 2 diabetes [5, 6] and stage 1 hypertension [7, 8], most cover only single aspect of health-promoting lifestyles, mainly physical activity and nutrition intake [5–8]. Compared to these one-sided estimations, the Health-Promoting Lifestyle Profile II (HPLP-II) provides a multidimensional assessment of health-promoting lifestyle. By elucidating any relationships of the prevalence of obesity and estimated common chronic diseases, including estimated anemia, estimated chronic kidney disease (CKD), and high cardiovascular disease (CVD) risk with participants’ HPLP-II and imbalances in subscales, particular dimensions and communities that require focus can be defined. Additionally, the HPLP-II is easy to assess; self-assessment and attempts at change can be done by the general public.

The purposes of study were to (1) investigate if there are any relationships between obesity and the three estimated common chronic diseases (anemia, CKD, and high CVD risk) and the HPLP-II in Hong Kong adults and (2) determine if there are any significant differences in lifestyle between individuals with and without estimated chronic diseases.

## Methods

### Study design and participants

This study was a secondary analysis of a data set in which the HPLP-II was considered an exposure variable, while obesity and estimated chronic disease risks were the two outcome variables. Data were obtained from a lifestyle study carried out by the Chinese University of Hong Kong.

The original study’s participants were recruited voluntarily through advertisements posted on the school campus. Eligible participants were community-dwelling Chinese adults aged 18–80 years without eye diseases that affect retinal photographs. Participants were asked to complete the HPLP-II questionnaire [9], and on-site tests were conducted to record demographic variables (age, sex, etc.), perform body measurements, and take retinal images. Written informed consent were obtained from all participants before the start of the study.

### Sample size calculation

We adopted the sample size calculation approach by Peduzzi et al. (1996) for logistic regression analysis, in which the sample size was calculated assuming *p*, the proportion of the population with chronic diseases, to be 28.4% [3] and the number of predictors, *K*, to be six, equaling the number of subscales in the HPLP-II. Based on the above assumptions and eq. *N* = 10 *K*/*p*, a sample size of 264 was needed assuming 20% of the surveys were incomplete [10].

### Data collection

#### HPLP-II questionnaire

Although many instruments are available for health behaviors measurement, most of them cover only a single aspect of health-promoting lifestyles [11]. Compared to these tools, the HPLP-II provides a multidimensional assessment of health- promoting lifestyle with high reliability and validity [12].

HPLP-II is a 52-item questionnaire comprising a total scale and six subscales scores in the six theorized dimensions, including health responsibility, physical activity, nutrition, spiritual growth, interpersonal relations, and stress management. Each item in the questionnaire is responded to using a four-point Likert scale scored as 1, 2, 3, and 4 for responses Never (N), Sometimes (S), Often (O), and Routinely (R), respectively [9]. In accordance with a previous study, responses with a mean score of ≥2.50 were considered positive [13].

#### Obesity and chronic disease risks

The first study outcome, obesity, was defined by body mass index (BMI) and waist-hip ratio (WHR) calculated using clinical data measured during the on-site physical assessment; the second outcome variable, estimated chronic diseases, was estimated using automatic retinal image analysis (ARIA).

##### On-site physical assessment for obesity

We collected physical data including age, sex, height, weight, waist circumference, and hip-circumference during the on-site physical assessment. Obesity indicators, WHR and BMI [14], were calculated using physical data. BMI was calculated by weight (kg)/(height [m])^2^ where the weights and heights measurements of the participants were recorded to the nearest 0.1 kg and 0.01 m, respectively. Using the Asian-specific obesity classification system, participants were categorized into the underweight/normal weight group (< 23 kg/m^2^) and the overweight/obese group (≥23 kg/m^2^) [15]. WHR was measured as waist circumference (cm)/hip circumference (cm). Overweight/obese was defined as a WHR > 0.90 and > 0.85 for men and women, respectively [16, 17].

##### ARIA to estimate chronic disease

A beta-testing approach of the ARIA method was used to estimate the chronic disease parameters, including total cholesterol (TC), low-density lipoprotein cholesterol (LDL-C), high-density lipoprotein cholesterol (HDL-C), hemoglobin, and estimated glomerular filtration rate (eGFR), to determine the estimated chronic disease for estimated anemia, estimated CKD, and estimated CVD risks. The working fundamentals of ARIA are based on retinal characteristics. The analysis extracts retinal risk factors related to cardiovascular changes from participant’s retinal images.

To estimate chronic diseases, method has been developed [18] for the evaluation of cerebral vessel conditions by analyzing retinal photographs pixel by pixel and produce measurements on retinal vascular parameters such as exudates, hemorrhages, and new vessels to achieve an overall evaluation of the parameters. Finally, the retinal information is converted using a beta-testing methodology into quantifiable and analyzable indicators, including TC, LDL-C, HDL-C, hemoglobin, and eGFR. The detailed procedure of ARIA can be found in Zee [18].

Retinal images were taken using Digital Non-Mydriatic Retinal Cameras CR-2 (Canon U.S.A., Inc.).

##### Determination of the risks of chronic diseases

Based on the estimated disease parameters by ARIA, the chronic diseases in the study population were estimated and then classified using the International Classification of Diseases.

##### Risk of anemia

Subjects with estimated hemoglobin levels < 11.0 g/dL were considered anemic [19].

##### Risk of CKD

Estimated CKD is suggested as a urinary albumin-to-creatinine ratio of > 30 mg/g or an eGFR of < 60 mL/min/1.73 m^2^ [20, 21].

##### Risk of CVD

The ratio of estimated TC and estimated HDL-C (TC/HDL-C), also known as the atherogenic or Castelli index, is a reliable indicator of CVD risk [22]. A high TC/HDL-C ratio indicates greater vascular risk, which may due to an increase in the atherogenic component or a decrease in the anti-atherosclerotic trait [23]. The cutoff levels of the TC/HDL-C ratio for the high risk of cardiovascular events are suggested to be > 5.0 for men and > 4.5 for women [24].

### Statistical analysis

The proportion test was used to compare the prevalence of estimated chronic diseases in the study population versus population data in Hong Kong to check for representativeness. An independent sample t-test was used to identify any association between obesity, the estimated chronic diseases, and a health-promoting lifestyle, while Welch’s t-test was used for variables with unequal variances. Binary logistic regression models were built to describe the patterns of association while controlling for potential confounding variables, including age and sex, in all models. The effect of explanatory variables on the response was evaluated by model parameters. The backward selection was used to obtain the final regression model.

The alpha level (*α*) was set at 0.05, and *p*-values smaller than *α* were considered statistically significant. Since this was an exploratory study, *p*-values smaller than 0.1 were considered marginally significant to provide a more conservative discussion about the generated statistics.

All data were analyzed using the Statistical Package for Social Sciences, version 24 (SPSS, IBM Corp., Armonk, NY).

## Results

### Sample characteristics

A total of 270 Chinese adults in Hong Kong were included. Demographic information and the prevalence of obesity and estimated chronic diseases for the study sample are presented in Table 1. Of the 270 respondents, 56 (20.7%) were male, and 214 (79.3%) were female. The participants’ age ranged from 24 to 79 years, with a mean age of 59.61 years. More than half of the participants were overweight or obese, in particular, 54.4% had overall obesity and 51.9% had central obesity. The prevalence of estimated anemia and estimated CKD was 25.2 and 10.4%, respectively, while 11.9% had a higher risk of CVD.

**Table 1:** Demographic characteristics and the prevalence of obesity and chronic diseases of the sample (*n* = 270)

	N	Percentage
Age, years (mean ± SD)	59.61 ± 1.15	
Sex		
Male	56	20.7
Female	214	79.3
Overweight or obese		
Overall adiposity (BMI)^†^	147	54.4
Abdominal adiposity (WHR)^†^	140	51.9
Estimated Chronic diseases		
Anemia^†^	68	25.2
Chronic kidney disease^†^	28	10.4
High cardiovascular disease risk^†^	32	11.9

†For BMI, a ‘Yes’ response referred to participants with a BMI ≥ 23 kg/m^2^; for WHR, ‘Yes’ was defined as a WHR > 0.90 and > 0.85 for men and women, respectively; for estimated anemia, subjects with a hemoglobin level < 11.0 g/dL were considered in the ‘Yes’ group; for estimated chronic kidney disease, a ‘Yes’ response was defined as an eGFR < 60 mL/min/1.73 m^2^; for Risk of cardiovascular disease, individuals with a estimated TC/HDL-C ratio > 5.0 for men and > 4.5 for women were categorized into the “high-risk” group in the table, while the others belonged to the “average-risk” group.

*SD* standard deviation, *BMI* body mass index, *WHR* waist-hip ratio, *eGFR* estimated glomerular filtration rate, *TC/HDL-C* total cholesterol/high-density lipoprotein cholesterol

### Variations in HPLP-II scores within sex, obesity, and chronic diseases categories

As shown in Table 2, the mean scores for all HPLP-II dimensions indicated positive responses (≥2.5). The highest values were seen in the spiritual growth and interpersonal relations subscales with the same score of 2.78 (± 0.53 for SG and ± 0.47 for IR), while the lowest was in the physical activity sub-dimension (2.50 ± 0.58).

**Table 2:** HPLP-II dimensions for all respondents (mean ± SD)^*^

Categories							
	OS	HR	PA	NT	SG	IR	SM
Individual average	2.67 ± 0.40	2.51 ± 0.48	2.50 ± 0.58	2.77 ± 0.44	2.78 ± 0.53	2.78 ± 0.47	2.66 ± 0.47
Sex							
Female	2.67 ± 0.41	2.54 ± 0.49	2.48 ± 0.57	2.79 ± 0.43	2.77 ± 0.55	2.78 ± 0.48	2.64 ± 0.48
Male	2.67 ± 0.36	2.40 ± 0.43	2.60 ± 0.62	2.67 ± 0.45	2.83 ± 0.42	2.79 ± 0.41	2.72 ± 0.44
*p*-value	0.984	0.059	0.152	0.067	0.388	0.828	0.279
Overweight or obese - overall adiposity (BMI)							
Yes	2.67 ± 0.41	2.51 ± 0.49	2.48 ± 0.59	2.74 ± 0.41	2.80 ± 0.57	2.82 ± 0.46	2.67 ± 0.52
No	2.66 ± 0.38	2.50 ± 0.47	2.53 ± 0.57	2.80 ± 0.47	2.75 ± 0.47	2.74 ± 0.47	2.64 ± 0.41
*p*-value	0.840	0.865	0.518	0.219	0.407	0.172	0.676
Overweight or obese - abdominal adiposity (WHR)							
Yes	2.67 ± 0.43	2.47 ± 0.50	2.47 ± 0.60	2.77 ± 0.42	2.79 ± 0.58	2.78 ± 0.49	2.69 ± 0.51
No	2.67 ± 0.36	2.55 ± 0.46	2.53 ± 0.56	2.77 ± 0.45	2.77 ± 0.46	2.78 ± 0.44	2.62 ± 0.43
*p*-value	0.890	0.199	0.391	0.952	0.700	0.971	0.190
Estimated anemia							
Yes	2.66 ± 0.41	2.47 ± 0.51	2.54 ± 0.62	2.74 ± 0.44	2.75 ± 0.53	2.75 ± 0.45	2.68 ± 0.51
No	2.67 ± 0.39	2.52 ± 0.47	2.49 ± 0.57	2.78 ± 0.43	2.79 ± 0.53	2.79 ± 0.47	2.65 ± 0.46
*p*-value	0.760	0.430	0.582	0.576	0.579	0.594	0.666
Estimated chronic kidney disease							
Yes	2.63 ± 0.36	2.52 ± 0.42	2.41 ± 0.48	2.79 ± 0.41	2.67 ± 0.48	2.76 ± 0.47	2.62 ± 0.53
No	2.67 ± 0.40	2.51 ± 0.49	2.51 ± 0.59	2.77 ± 0.44	2.79 ± 0.53	2.78 ± 0.47	2.66 ± 0.47
*p*-value	0.611	0.847	0.353	0.816	0.261	0.807	0.678
Estimated cardiovascular disease							
High risk	2.65 ± 0.41	2.54 ± 0.53	2.41 ± 0.59	2.69 ± 0.44	2.75 ± 0.54	2.80 ± 0.50	2.67 ± 0.51
Average risk	2.67 ± 0.39	2.50 ± 0.47	2.51 ± 0.58	2.78 ± 0.44	2.78 ± 0.52	2.78 ± 0.47	2.65 ± 0.47
*p*-value	0.759	0.670	0.335	0.312	0.768	0.797	0.876

^*^t-test of the HPLP-II dimensions for participants with and without chronic diseases. All responses were reported as means ± SD

*HPLP-II* Health-Promoting Lifestyle Profile II, *SD* standard deviation, *OS* overall HPLP-II score, *HR* Health Responsibility, *PA* Physical Activity, *NT* Nutrition, *SG* Spiritual Growth, *IR* Interpersonal Relations, *SM* Stress Management

Both sexes had an overall score of 2.67 (± 0.41 for women and ± 0.36 for men). Women had significantly higher scores for health responsibility (*p* = 0.059) and nutrition (*p* = 0.067) than men. Conversely, their scores for physical activity, spiritual growth, interpersonal relations, and stress management were lower than for men; however, the differences were not significant.

Regarding the obesity outcomes, BMI and WHR, no significant differences were noted between the normal and overweight/obese subjects in all the HPLP-II dimensions. Overall, HPLP-II scores of overweight/obese respondents were generally greater than or equal to that of normal weight respondents. The overweight/obese group defined by BMI reported higher scores in all HPLP-II subscales than the normal-weight group except for physical activity and nutrition. Similarly, participants with a WHR corresponding to the overweight/obese category claimed to have higher or equal scores in all dimensions except health responsibility and physical activity.

Meanwhile, for the estimated chronic disease outcomes, the study revealed that patients with estimated chronic diseases generally had lower HPLP-II scores than those without chronic diseases; however, the difference was not significant (Table 2).

### Predictive power of HPLP-II subscales for obesity and estimated chronic diseases

Table 3 summarizes the results of the final regression models. After controlling for relevant variables, the results of the logistic regression model showed that nutrition and interpersonal relations were significant independent predictors of BMI (*p* = 0.017 and 0.016, respectively). People with a higher score in the nutrition subscale (adjusted odds ratio [AOR] = 0.41, 95% confidence interval [CI] = 0.23–0.86) were more likely to have a lower BMI, while 1-unit increased in interpersonal relations resulted in a 0.76 increase in BMI (AOR = 2.14, 95% CI = 1.15–3.96).

**Table 3:** Logistic regression outcomes of obesity and chronic disease risks with HPLP-II subscales as predictors

Categories	AOR	95% CI	*p*-value
Overweight or obese			
Overall adiposity (BMI)			
IR	2.14	1.15 to 3.96	0.016
NT	0.41	0.23 to 0.86	0.017
Age	1.04	1.01 to 1.07	0.009
Abdominal adiposity (WHR)			
PA	0.50	0.28 to 0.87	0.015
SM	2.07	1.04 to 4.14	0.038
Estimated HDL-C	1.67	1.01 to 2.75	0.046
Age	1.06	1.03 to 1.10	< 0.001
Estimated anemia			
Sex (ref. Female)	3.33	1.58 to 7.00	0.002
Estimated TC	0.61	0.49 to 0.76	< 0.001
Estimated HDL-C	0.44	0.23 to 0.84	0.012
Estimated LDL-C	0.63	0.50 to 0.80	< 0.001
Estimated chronic kidney disease			
SG	0.24	0.05 to 1.17	0.077
IR	5.06	0.88 to 28.98	0.069
Estimated HDL-C	0.11	0.04 to 0.30	< 0.001
Estimated hemoglobin	0.48	0.34 to 0.67	< 0.001

*HPLP-II* Health-Promoting Lifestyle Profile II, *AOR* adjusted odds ratio, *CI* confidence interval, *BMI* body mass index, *NT* Nutrition, *IR* Interpersonal relations, *WHR* waist-hip ratio, *PA* Physical activity, *SM* Stress management, *LDL-C* low-density lipoprotein cholesterol, *TC* total cholesterol, *HDL-C* high-density lipoprotein cholesterol; SG, Spiritual growth

The multivariate analysis for WHR revealed a significant association between stress management with WHR (*p* = 0.038). A higher score in stress management (AOR = 2.07, 95% CI = 1.04–4.14) was associated with a higher WHR, while participants with a higher score in physical activity (AOR = 0.50, 95% CI = 0.28–0.87) were significantly more likely to have a lower WHR (*p* = 0.015).

The results of the binary regression model showed that spiritual growth (*p* = 0.077) and interpersonal relations (*p* = 0.069) were significant predictors of estimated CKD. People with a higher score in the interpersonal relations subscale (AOR = 5.06, 95% CI = 0.88–28.98) were more likely to have estimated CKD, while a higher score in spiritual growth (AOR = 0.24, 95% CI = 0.05–1.17) was negatively associated with estimated CKD.

No significant predictive power for HPLP-II indicators regarding estimated anemia and CVD risk was found (all *p*-values > 0.10).

## Discussion

The study investigated the relationship between obesity, estimated common chronic diseases and the HPLP-II. Overall, the results revealed a significant association of obesity, and estimated CKD, the two common chronic diseases, with HPLP-II. Respondents with estimated chronic diseases had lower HPLP-II scores than healthy individuals; however overweight/obese individuals scored higher than or equal to the normal-weight participants. The finding also showed a sex-difference trend in the health responsibility and nutrition subscales of the HPLP-II. No significant differences were observed between the prevalence of overall obesity, anemia and chronic kidney disease in the study sample and the general population (Additional file 1).

Our regression models showed that HPLP-II subscales contributed to the prediction of obesity and estimated CKD. Among the obesity outcomes, physical activity and nutrition intake appeared to be protective factors for obesity. Persons with better nutrition knowledge and dietary intake (i.e., higher score for Nutrition) or more physically active tended to have a lower risk for overweight/obesity than those who scored lower in these subitems. The results were consistent with previous research that reported beneficial effects of healthy dietary behavior and a more active lifestyle on weight control [11, 25–27]. Conversely, better stress management and interpersonal relations were associated with a higher probability of obesity. These results seem to partially contradict the previous findings that healthy social relationships would have beneficial effects on obesity [28], while stress was positively correlated with obesity [29, 30]. We found an inverted U-shaped relationship between stress management and interpersonal relations and the obesity index using curve estimation (Additional files 2 and 3). As BMI increased, the interpersonal relations score increased; however, the rise in the interpersonal relations score was only to the point of a BMI around 23 kg/m^2^, the borderline of normal weight, beyond which an increase in BMI led to a reduction in the interpersonal relations score (Additional file 2). A similar result was found regarding the relationship between stress management and WHR (Additional file 3). Our results partially support previous findings and emphasize the significance of stress management and interpersonal relations for obesity.

The regression model of estimated CKD revealed a negative correlation between spiritual growth and the risk of CKD, and a positive correlation between estimated CKD and interpersonal relations. Some studies have determined the spiritual needs of patients with CKD regarding their treatment plans [31, 32]. However, studies of spiritual growth and interpersonal relations in the context of the risk of CKD are limited. Our exploratory results provide a potential research topic for further investigation on the role of spiritual well-being and interpersonal relations in the development of CKD.

Our study was unable to find a significant relationship between lifestyle, estimated anemia, and the risk of CVD. Studies that have investigated the predictive power of HPLP-II for chronic diseases are lacking. Therefore, further studies on other common chronic diseases, such as hypertension and diabetes, are recommended. The regression model of anemia also provided some new insights, i.e., the serum lipid profile including TC, HDL-C, and LDL-C was significantly different between anemic and non-anemic individuals [33]. Studies that investigate the relationship between cholesterol and anemia are recommended to determine the underlying mechanisms of this finding.

Apart from the results of the regression model, the finding that the HPLP-II scores of healthy participants tended to be higher than those of participants with chronic diseases is supported by the studies of Cao et al. and Thanavaro et al. [34, 35]. An explanation for this finding is that individuals with higher scores are likely to possess positive health behaviors which are inversely correlated with obesity and multiple chronic diseases [36–38].

Our results supported the finding that sex differences exist in health-promoting lifestyles [39, 40]. Our data confirmed that women had significantly better health responsibility and nutrition than men. The subscales in which women surpassed men were consistent with the reports by Johnson and Wei et al. [39, 40]. Sex-difference trends have also been demonstrated in the prevalence of obesity over the last few decades in the Asian population. Furthermore, disease vulnerability and an awareness of health differ according to sex; thus, sex-specific public health interventions may be essential in the future [41]. As for women, postmenopausal status is known to be a key risk factor for metabolic alterations and the development of chronic diseases [42]. Our study did not account for menopausal status; therefore, further studies that will consider menopausal status as a factor in the relationship between lifestyle, sex, and chronic diseases are recommended. Our results underscore the necessity of sex-sensitive research and approaches for improved preventive programs for various chronic diseases.

Our results suggest an association between lifestyle and health conditions, implying the possibility to control or alleviate the prevalence of chronic diseases by the modification of health behaviors. These findings could arouse concern about lifestyle behaviors and promote self-assessment of health-promoting lifestyles to the general public. Health promotion and disease prevention programs could be focused on particular dimensions defined by imbalances in the HPLP-II subscales, such as the physical inactivity issue in society reflected by the lowest score in the physical activity subscale.

Some limitations should be considered. The chronic disease statuses were estimated from the beta-testing version of the ARIA for chronic diseases parameters. The study used a noninvasive detection approach based on retinal characteristics, the ARIA, instead of traditional clinical data. Nevertheless, using several disease-specific retinal characteristics, ARIA provides insights into the clinical interpretation of risk estimation and diseases diagnosis [43]. For instance, in the development of CKD, the occurrence of subclinical renal microvascular abnormalities is reflected by specific retinal microvascular abnormalities, including smaller retinal arterioles, larger retinal venules, and the presence of retinopathy [44] which is defined as the presence of one or more microaneurysms or retinal hemorrhages [45]. Previous studies have shown that retinal characteristics not only add value to the clinical differentiation between diseases statuses, such as stroke and CKD [43, 44, 46–48], but can also be ascertained reliably by standardized photographic grading methods using computer-assisted quantification of the retinal characteristics, supporting the validity of the associations between retinal characteristics and diseases [48].

Second, due to the limited resources, the sample size is relatively small. The true differences may not be demonstrated and it may limit inferences about causal relationships between health conditions and health-promoting lifestyles. With greater resources, a longitudinal design with larger sample size could be applied to investigate the causal relationship between health-promoting lifestyles and chronic diseases. For the same reason that our resources are limited, participants in this study could not be randomly sampled in all 18 districts in Hong Kong. However, our samples were recruited voluntarily from the general public and they came from community-dwelling backgrounds. Moreover, the prevalence of anemia, CKD and obesity in the sample were not significantly different from those in the general population (Additional file 1); therefore, the sample representativeness was considered acceptable for this study. Further research using a random sampling method is also recommended to have a more representative cohort.

To our knowledge, this study is the first to examine the relationship between obesity, common chronic diseases, and the HPLP-II in Hong Kong Chinese adults, shedding light on the importance of lifestyle factors in East Asian People as they become more westernized. Additionally, instead of using traditional assessment methods like blood tests, this study used an innovative noninvasive diagnostics approach, ARIA, which is easy to perform and useful in the public health management setting.

## Conclusion

In summary, this study demonstrated the physical inactivity issue through the imbalanced HPLP-II subscale score. Moreover, our findings, which revealed lower HPLP-II score in patients with estimated chronic diseases than in healthy individuals, suggest that people with low HPLP-II scores should be concerned about their health. An association between obesity, estimated CKD, and health-promoting lifestyles was noted, indicating a potential role for health-promoting lifestyles in alleviating the risk of obesity and CKD. The study also provided new insights into the relationship between the HPLP-II and other common chronic diseases that warrants further study. Healthcare providers should be involved actively in improving lifestyle factors and modifying barriers in the promotion of health among Hong Kong people of all ages.

## Supplementary Information

**Additional file 1.** Proportion test results of diseases’ prevalence in sample and the general population. No significant difference was observed between the prevalence of overall obesity in the study sample and the general population of Hong Kong retrieved from the PHS [2]. Similarly, there was no significant difference between the prevalence of estimated anemia in sample and the worldwide reported by WHO [49]. The prevalence of estimated CKD in sample was in the same range as that in Asia [50].

**Additional file 2.** Quadratic effect of the Interpersonal Relationship score predicting BMI. The curve estimation showed an inverted U-shaped relationship between interpersonal relations and BMI. As the BMI increased, the interpersonal relations score increased; beyond a BMI of approximately 23 kg/m^2^, the borderline of normal weight, an increase in BMI was associated with a reduction in the interpersonal relations score.

**Additional file 3.** Quadratic effect of the Stress Management score predicting WHR. The curve estimation showed an inverted U-shaped relationship between stress management and WHR. As the WHR increased, the stress management score increased; beyond a WHR of approximately 0.9, the borderline of normal weight, an increase in WHR was associated with a reduction in the stress management score.

## Data Availability

The datasets used and/or analyzed in the current study are available for fulfilling partially of a Master Degree Program of YSL. The datasets generated and/or analysed during the current study are not publicly available due to confidentiality requirements.

## Abbreviations

AOR: Adjusted odds ratio
ARIA: Automatic retinal image analysis
BMI: Body mass index
CI: Confidence interval
CKD: Chronic kidney disease
CVD: Cardiovascular disease
eGFR: Estimated glomerular filtration rate
HDL-C: High-density lipoprotein cholesterol
HPLP-II: Health-Promoting Lifestyle Profile II
LDL-C: Low-density lipoprotein cholesterol
NCD: Non-communicable disease
PHS: Population Health Survey
TC: Total cholesterol
WHR: Waist-hip ratio
