# Supplementary material for: Association between obesity, common chronic diseases and health promoting lifestyle profiles in Hong Kong adults: a cross-sectional study
Source: BMC Public Health. 2020 Oct 28;20:1624. doi: 10.1186/s12889-020-09726-x (PMC7594285; doi:10.1186/s12889-020-09726-x)
Supplement: Supplementary file 1 — Additional file 1. Proportion test results of diseases’ prevalence in sample and the general population. No significant difference was observed between the prevalence of overall obesity in the study sample and the general population of Hong Kong retrieved from the PHS [2]. Similarly, there was no significant difference between the prevalence of estimated anemia in sample and the worldwide reported by WHO [49]. The prevalence of estimated CKD in sample was in the same range as that in Asia [50]. [file 12889_2020_9726_MOESM1_ESM.docx]

**Additional file 1:** MS Word document (.docx)

**Title:** Proportion test results of diseases’ prevalence in sample and the general population

|  | **Prevalence of Diseases (%)** | |  |
| --- | --- | --- | --- |
|  | **Sample** | **General population** | **p-value** |
| **Overweight or obesity**^†^ | 54.4 | 50^a^ | 0.148 |
| **Anemia** | 25.2 | 24.8^b^ | 0.879 |
| **Chronic kidney disease** | 10.4 | 10 – 18^c^ | - |

^†^Prevalence of overweight/obesity in sample and the general population in this table was both defined by a BMI ≥ 23.0 kg/m^2^.

*Note.* The prevalence of overweight/obesity in the general population of Hong Kong was retrieved from the 2nd Population Health Survey by HK Department of Health (2017)^a^. The prevalence of the worldwide anemia was from WHO Global Database on Anaemia Geneva by World Health Organization (2008)^b^, while the prevalence range of CKD in Asia was retrieved from a systematic review by Khan et al. (2018)^c^.
