# Supplementary material for: Association between obesity, common chronic diseases and health promoting lifestyle profiles in Hong Kong adults: a cross-sectional study
Source: BMC Public Health. 2020 Oct 28;20:1624. doi: 10.1186/s12889-020-09726-x (PMC7594285; doi:10.1186/s12889-020-09726-x)

**Additional file 2:** MS Word document (.docx)

**Title:** Quadratic effect of the Interpersonal Relations score predicting BMI


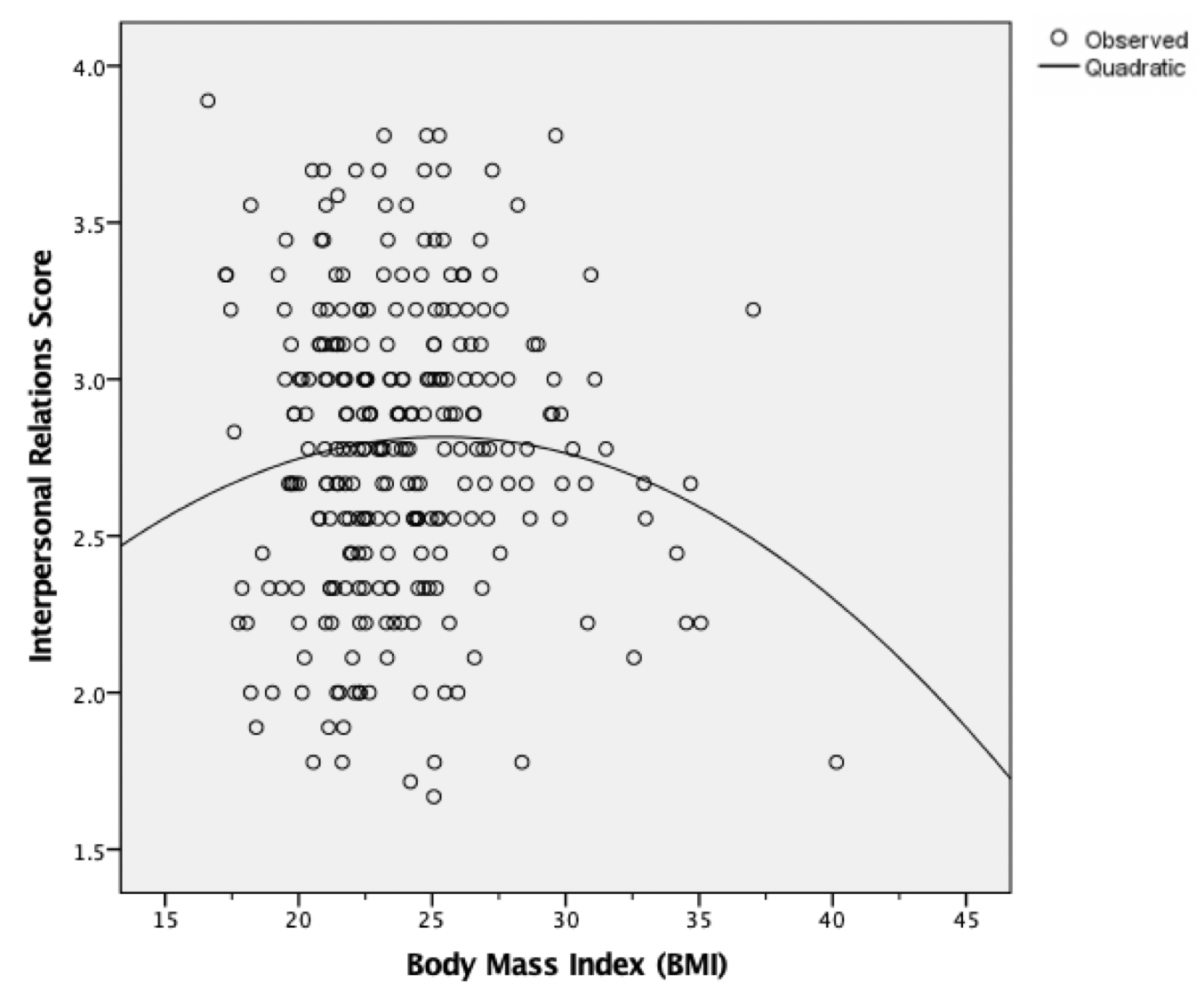

Supplement: Supplementary file 2 — Additional file 2. Quadratic effect of the Interpersonal Relationship score predicting BMI. The curve estimation showed an inverted U-shaped relationship between interpersonal relations and BMI. As the BMI increased, the interpersonal relations score increased; beyond a BMI of approximately 23 kg/m2, the borderline of normal weight, an increase in BMI was associated with a reduction in the interpersonal relations score. [file 12889_2020_9726_MOESM2_ESM.docx]
