# Supplementary material for: Association between obesity, common chronic diseases and health promoting lifestyle profiles in Hong Kong adults: a cross-sectional study
Source: BMC Public Health. 2020 Oct 28;20:1624. doi: 10.1186/s12889-020-09726-x (PMC7594285; doi:10.1186/s12889-020-09726-x)

**Additional file 3:** MS Word document (.docx)

**Title:** Quadratic effect of the Stress Management score predicting WHR


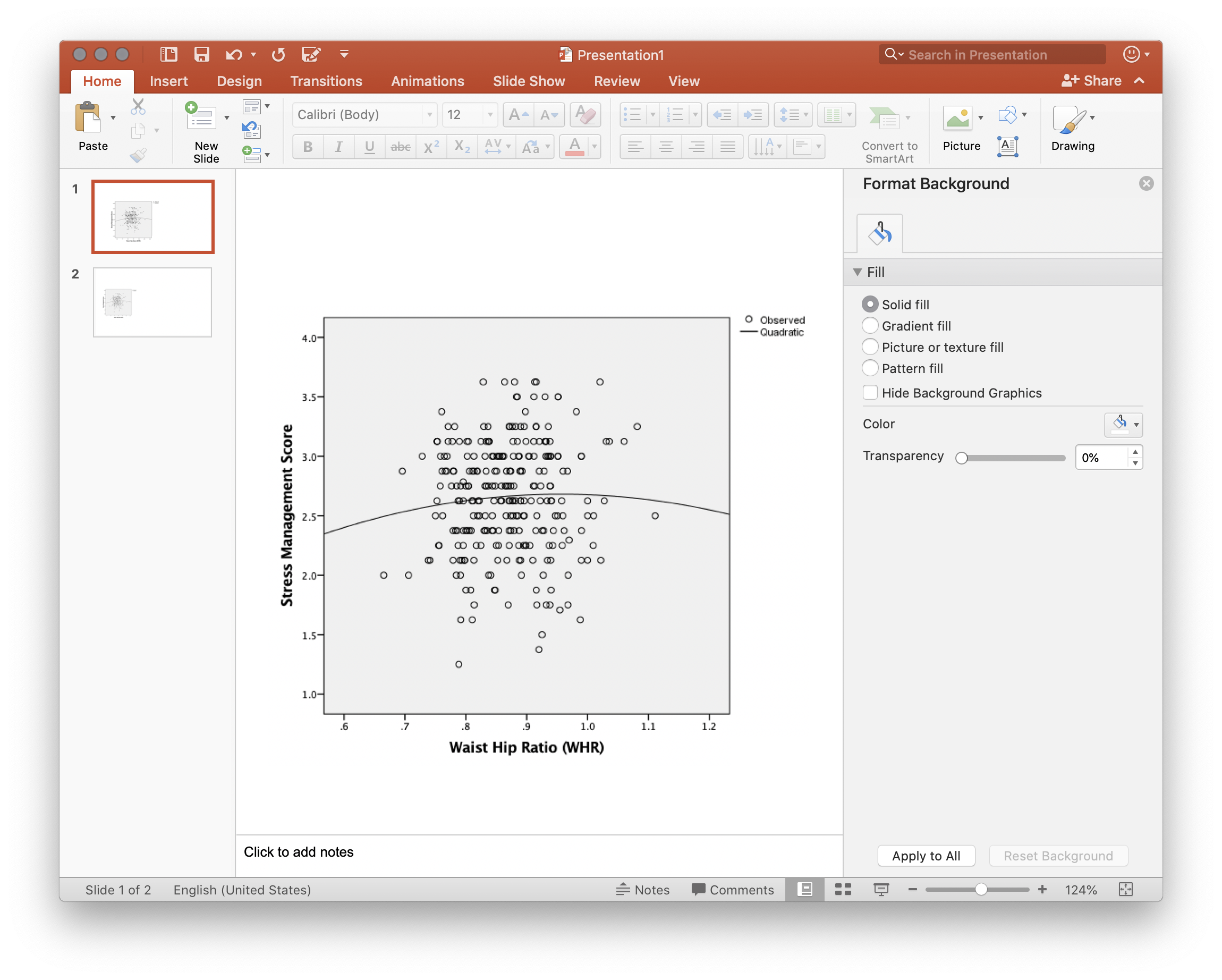

Supplement: Supplementary file 3 — Additional file 3. Quadratic effect of the Stress Management score predicting WHR. The curve estimation showed an inverted U-shaped relationship between stress management and WHR. As the WHR increased, the stress management score increased; beyond a WHR of approximately 0.9, the borderline of normal weight, an increase in WHR was associated with a reduction in the stress management score. [file 12889_2020_9726_MOESM3_ESM.docx]
